# Differential sensitivity of melanoma cell lines with *BRAF*^*V600E *^mutation to the specific Raf inhibitor PLX4032

**DOI:** 10.1186/1479-5876-8-39

**Published:** 2010-04-20

**Authors:** Jonas N Søndergaard, Ramin Nazarian, Qi Wang, Deliang Guo, Teli Hsueh, Stephen Mok, Hooman Sazegar, Laura E MacConaill, Jordi G Barretina, Sarah M Kehoe, Narsis Attar, Erika von Euw, Jonathan E Zuckerman, Bartosz Chmielowski, Begoña Comin-Anduix, Richard C Koya, Paul S Mischel, Roger S Lo, Antoni Ribas

**Affiliations:** 1 Department of Medicine, Division of Hematology/Oncology, University of California Los Angeles (UCLA), Los Angeles, CA, USA; 2 Department of Surgery, Division of Surgical Oncology, UCLA, Los Angeles, CA, USA; 3 Department of Medicine, Division of Dermatology, UCLA, Los Angeles, CA, USA; 4 Department of Pathology and Laboratory Medicine, UCLA, Los Angeles, CA, USA; 5 The Broad Institute of MIT and Harvard, Cambridge, MA USA; 6 Departments of Medical and Pediatric Oncology and Center for Cancer Genome Discovery, Dana-Farber Cancer Institute, Harvard Medical School, Boston, MA, USA; 7 Jonsson Comprehensive Cancer Center at UCLA, Los Angeles, CA, USA; 8 Current address: Department of Systems Biology, Molecular Immune Regulation at the Center for Biological Sequence Analysis, Technical University of Denmark, Lyngby, Denmark

## Abstract

Blocking oncogenic signaling induced by the *BRAF*^V600E ^mutation is a promising approach for melanoma treatment. We tested the anti-tumor effects of a specific inhibitor of Raf protein kinases, PLX4032/RG7204, in melanoma cell lines. PLX4032 decreased signaling through the MAPK pathway only in cell lines with the *BRAF*^V600E ^mutation. Seven out of 10 *BRAF*^V600E ^mutant cell lines displayed sensitivity based on cell viability assays and three were resistant at concentrations up to 10 μM. Among the sensitive cell lines, four were highly sensitive with IC_50 _values below 1 μM, and three were moderately sensitive with IC_50 _values between 1 and 10 μM. There was evidence of MAPK pathway inhibition and cell cycle arrest in both sensitive and resistant cell lines. Genomic analysis by sequencing, genotyping of close to 400 oncogeninc mutations by mass spectrometry, and SNP arrays demonstrated no major differences in *BRAF *locus amplification or in other oncogenic events between sensitive and resistant cell lines. However, metabolic tracer uptake studies demonstrated that sensitive cell lines had a more profound inhibition of FDG uptake upon exposure to PLX4032 than resistant cell lines. In conclusion, *BRAF*^V600E ^mutant melanoma cell lines displayed a range of sensitivities to PLX4032 and metabolic imaging using PET probes can be used to assess sensitivity.

## Background

Improved knowledge of the oncogenic events in melanoma indicates that a majority of mutations activate the mitogen-activated protein kinase (MAPK) pathway [1,2]. The most frequent mutation in the MAPK pathway is in the *BRAF *gene, present in 60-70% of malignant melanomas [3]. *NRAS *mutations occur in approximately 15% of melanomas [1,4,5] and are mutually exclusive with *BRAF *mutations [6,7]. The majority of mutations in *BRAF *are accounted for by a single nucleotide transversion from thymidine to adenosine leading to a substitution of valine by glutamic acid at position 600 (termed *BRAF*^V600E^) [3,4,8], which leads to a 500-fold increase in activity compared to the wild type protein kinase [8].

PLX4032 (also known as RG7204) was developed as a specific inhibitor of Raf. It is an analogue of the pre-clinically tested PLX4720 [9]. PLX4720 inhibits the mutated B-Raf kinase at 13 nM, while the wild type kinase requires tenfold higher concentration (160 nM) [9], thus predicting high specificity for *BRAF*^V600E ^mutant cell lines. The basis of this specificity for the mutated kinase is thought to be the preferential inhibition of the active conformation of B-Raf. In addition, its access to a Raf-selective pocket accounts for the selectivity against most other non-Raf kinases, which require concentrations 100 to 1000 times higher for kinase inhibition. The only exception is the breast tumor kinase (BRK), which is inhibited at 130 nM, a one-log difference compared to the V600E mutated B-Raf kinase [9].

In the current studies we analyzed a panel of human melanoma cell lines with defined oncogenic alterations for sensitivity to PLX4032. In addition, with a view to development of a biomarker to indicate response to targeted therapy, we investigated a non-invasive method of imaging resistance versus sensitivity *in vivo*. We describe that PLX4032 works differentially in melanoma cell lines with *BRAF*^V600E ^mutations and that the positron emission tomography (PET) tracer 2-fluoro-2-deoxy-D-glucose (FDG) can be used in non-invasive PET imaging to distinguish between sensitive and resistant cell lines.

## Materials and methods

### Reagents and cell lines

PLX4032 (also known as RG7204 or RO5185426) was obtained under a materials transfer agreement (MTA) with Plexxikon (Berkeley, CA) and dissolved in DMSO (Fisher Scientific, Morristown, NJ) to a stock concentration of 10 mM. SKMEL28 was obtained from American Type Culture Collection (ATCC, Rockville, MD), and the remaining human melanoma cell lines (M series) were established from patient's biopsies under UCLA IRB approval #02-08-067. Cells were cultured in RPMI 1640 with L-glutamine (Mediatech Inc., Manassas, VA) containing 10% (unless noted, all percentages represent volume to volume) fetal bovine serum (FBS, Omega Scientific, Tarzana, CA) and 1% penicillin, streptomycin, and amphotericin (Omega Scientific). All cell lines were mycoplasma free when periodically tested using a Mycoalert assay (Lonza, Rockland, ME).

### *BRAFV*^600E ^mutation analysis

Genomic DNA was extracted using FlexiGene DNA Kit (Qiagen, Valencia, CA) and the 200 bp region flanking the mutation site was amplified by PCR using Invitrogen online primer design (Invitrogen, Calsbad, CA) as described [10]. The PCR products were purified using QIAquick PCR Purification Kit (Qiagen), sequenced (Laragen Inc., Los Angeles, CA) and aligned with the *BRAF *gene (http://www.ncbi.nlm.nih.gov, accession no. NT_007914).

### Oncomap 3 core mass-spectrometric genotyping

Samples were run through OncoMap 3 which interrogates 396 somatic mutations across 33 genes. Whole genome amplified DNA at 5 ng/μl was used as input for multiplex PCR as described previously [11]. Single-basepair primer extension (iPLEX) was performed in a 2 μl reaction volume using iPLEX Gold single base extension enzyme (Sequenom, San Diego, CA). Products were resined and transferred to SpectroCHIPs for analysis by MALDI-TOF mass spectrometry [11]. All mutations were confirmed by direct sequencing of the relevant gene fragment.

### SNP array analysis

DNA extracted from the full panel of 13 human melanoma cell lines was hybridized onto Illumina Beadchip Human Exon 510S-Duo (Illumina Inc., San Diego, CA). DNA copy number was calculated using PennCNV (*) as described [12]. Eight of the cell lines (M202, M207, M229, M249, M255, M257, M263, M308) were additionally analyzed using Affymetrix GeneChip^® ^Human Mapping 250K Nsp Array (Affymetrix, Santa Clara, CA).

### Cell proliferation and viability assays

Melanoma cell lines were treated in triplicates with PLX4032 and parallel vehicle control in the given concentrations for 120 hours. Viable cells was measured using a tetrazolium compound [3-(4,5-dimethylthiazol-2-yl)-5-(3-carboxymethoxyphenyl)-2-(4-sulfophenyl)-2H-tetrazolium (MTS)-based colorimetric cell proliferation assay (Promega, Madison, WI). Cell line doubling time were determined from cell numbers measured in duplicates every 24 hours for a period of 9 to 12 days using a Vi-CELL series cell viability analyzer (Beckman Coulter). The doubling time in 24 hours was calculated by the formula 1/{[((logC2)-(logC1))×3.32]/T}, where C1 = the initial cell number, C2 = the final cell number, and T = 24 hours. The average of day 3, 4, 5 was used as the optimal doubling time for the given experimental condition.

### Phosphoflow staining

Cells were plated and treated with 1 μM PLX4032 or vehicle control for 1 or 20 hours, fixed in 1.6% paraformaldehyde (Electron Microscopy Sciences, Hatfield, PA), permeabilized in 4°C 100% methanol (Fisher Scientific) and stained with Alexafluor 647-conjugated human anti-phospho-Erk1/2 (T202/Y204, BD Biosciences, San Jose, CA) in sterile PBS (Mediatech Inc.) containing 0.5% albumin bovine serum and 0.01% sodium azide (both from Sigma-Aldrich, St. Louis, MO). Flow cytometry was performed on FACSCalibur or FACScan (BD Biosciences) and data was analyzed using FlowJo (Tree Star Inc, Asland, OR).

### Cell cycle analysis

Cells were treated with 1 μM PLX4032 and parallel vehicle control for 20 to 120 hours, fixed in 70% ethanol (Pharmco-Aaper, Shelbyville, KY), and then resuspended in sterile PBS containing 0.5% albumin bovine serum, 180 μL/ml propidium iodide staining solution (BD Biosciences) and 50 μg/mL ribonuclease A from bovine pancreas (Sigma-Aldrich). Flow cytometry was performed on FACSCalibur or FACScan and data was analyzed using FlowJo.

### Apoptosis analysis

Melanoma cell lines were treated with increasing concentrations of PLX4032, DMSO vehicle control, or 1 μM of staurosporine as a positive control, for 120 hours. Cells were trypsinized and transferred to FACS tubes and stained with Annexin V-FITC and propidium iodide following the manufacturer's instructions (BD Biosciences) and analyzed by flow cytometry using FACSCalibur as described [13].

### Western Blotting

Western blotting was performed as previously described [14]. Primary antibodies included p-Akt Ser473 and Thr308, Akt, p-S6K, S6K, p-S6 Ser235/236, S6, PTEN, p-ERK Thr204/205, ERK, p-AMPK, AMPK (all from Cell Signaling Technology, Danvers, MA), and α-actin (Sigma-Aldrich). The immunoreactivity was revealed by use of an ECL kit (Amersham Biosciences Co, Piscataway, NJ).

### *In vitro *metabolic tracer uptake assay

10^4 ^cells/well were plated on 0.001% poly-L-lysine (Sigma-Aldrich) pre-incubated filter bottom 96-well plates (multiscreen HTS GV 0.22 μm opaque, Millipore, Billerica, MA) and rested for 24 hours. 1 μM PLX4032 and parallel vehicle control were added in triplicates for 20 hours. Cells were incubated for 1 hour with 0.5 μCi with one of the three metabolic tracers with analogues used as PET tracers: 2-FDG [5,6-^3^H] (American Radiolabeled Chemicals Inc., St. Louis, MO) in glucose-free DMEM (Invitrogen), or 2'-Deoxy-2'-fluoroarabinofuranosylcytosine-[^3^H], and thymidine [methyl-^3^H] (FAC and thymidine, Moravek Biochemicals Inc., Brea, CA) in RPMI 1640. Extracellular metabolic tracer was washed off using a multiscreen HTS vacuum manifold system (Millipore). 100 μL scintillation fluid (Perkin Elmer, Waltham, MA) was added to each well and tritium count was measured on a 1450 microbeta trilux microplate (Perkin Elmer).

### *In vivo *microCT and microPET studies

Mice with established subcutaneous human melanoma xenografts were treated for 3 days with 100 mg/kg PLX4032 in corn oil or vehicle control twice daily by oral gavage. The last treatment was given one hour prior to intraperitoneal injection of 200 μCi [^18^F]-FDG, which was allowed to distribute in the tissues for 1 hour before microPET scanning as previously described [15].

### Statistical analysis

Continuous variables were compared using a paired Student's *t*-test with two-tailed P values.

## Results

### PLX4032 specifically blocks the MAPK pathway in melanoma cell lines with the *BRAF*^V600E ^mutation

**Figure 1:**
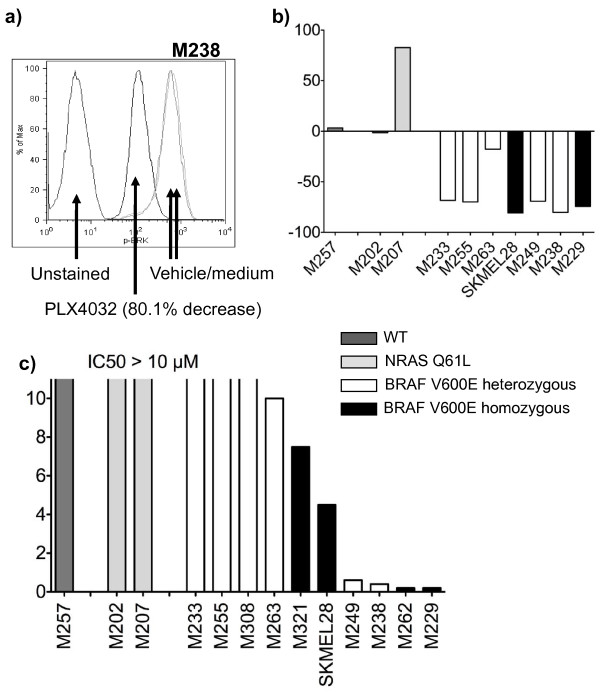
**PLX4032 modulation of the MAPK pathway and melanoma cell line viability**. Melanoma cell lines treated with 1 μM PLX4032 for 20 hours were stained with pErk antibody and analyzed by flow cytometry. a) Representative flow cytometry histogram showing the fluorescence intensity of pErk in cells treated with vehicle control or PLX4032. b) Comparison of percentage change in pErk for a panel of 10 melanoma cell lines with different *NRAS*/*BRAF *mutational status. c) *In vitro *cell viability upon culture with increasing concentrations of PLX4032 (from 0.001-10 μM) for 120 hours. Cell viability was determined using an MTS-based assay.

We tested the ability of PLX4032 to differentially block MAPK pathway signaling in a panel of human melanoma cell lines (Table 1) by quantitating the inhibition of phosphorylated Erk (pErk), a downstream target of B-Raf activity, using intracellular phosphospecific flow cytometry (Figure 1A). As expected, cell lines with *BRAF*^V600E ^mutation had a fast (detectable at 1 hour) and sustained (persistent at 20 hours, Figure 1B) inhibition of pErk, although one of the cell lines (M263) had lower inhibition of pErk than the rest. There was no pErk inhibition in two cell lines with *NRAS *Q61L mutation (M202 and M207) and a cell line wild type for both oncogenes (M257). In fact, there was a markedly increased pErk signal in one *NRAS *Q61L mutated cell line (M207), an observation consistent with data from others that has been attributed to loss of negative regulatory pathways [16,17] and enhanced signaling through C-Raf [18,19]. Therefore, PLX4032 inhibits MAPK pathway signaling specifically in cell lines that harbor the *BRAF*^V600E ^mutation.

**Table 1:** Genomic characterization, growth kinetics and sensitivity towards PLX4032 for a panel of human melanoma cell lines.

Cell Line	NRAS/BRAF	Number of *BRAF *Gene Copies	Other Oncogenic Events	Cell line doubling time (hours)	PLX4032 IC_50 _(μM)
M257	Wild type	3	*CDKN2A *R80	31.4	Not reached

M202	*NRAS *Q61L	2	*EGFR *amplification *CDKN2A *homozygous deletion	26.1	Not reached

M207	*NRAS *Q61L	2	*MITF *amplification *PTEN *heterozygous deletion	25.2	Not reached

M233	*BRAF*^V600E ^Heterozygous	3	*AKT1 *amplification *CCND1 *amplification *EGFR *amplification *CDKN2A *homozygous deletion *PTEN *homozygous deletion	29.6	Not reached

M255	*BRAF*^V600E ^Heterozygous	2	*AKT2 *amplification *CCND1 *amplification *EGFR *amplification *CDKN2A *homozygous deletion	48.6	Not reached

M308	*BRAF*^V600E ^Heterozygous	3	*MITF *amplification *AKT2 *amplification *EGFR *amplification *CDKN2A *heterozygous deletion	35.0	Not reached

M263	*BRAF*^V600E ^Heterozygous	2	*CDKN2A *heterozygous deletion	23.3	10

M321	*BRAF*^V600E ^Homozygous	2		34.1	7.5

SKMEL28	*BRAF*^V600E ^Homozygous	2	*EGFR *P753S *MITF *amplification *CCND1 *amplification *CDKN2A *heterozygous deletion *PTEN *heterozygous deletion	26.9	4.6

M229	*BRAF*^V600E ^Homozygous	4	MITF amplification AKT1 amplification *PTEN *heterozygous deletion	27.9	0.2

M238	*BRAF*^V600E ^Heterozygous	2	*CDKN2A *homozygous deletion *PTEN *heterozygous deletion	28.1	0.7

M249	*BRAF*^V600E ^Heterozygous	3	MITF amplification AKT2 amplification *PTEN *homozygous deletion	21.2	0.8

M262	*BRAF*^V600E ^Homozygous	2	AKT1 E17K AKT1 amplification *EGFR *amplification *CDKN2A *homozygous deletion	47.4	0.1

### Differential sensitivity to PLX4032 in *BRAF*^V600E ^mutated melanoma cell lines

Melanoma cell lines with different *NRAS*/*BRAF *mutational status were treated *in vitro *with a range of concentrations of PLX4032 for 5 days. The three cell lines without *BRAF*^V600E ^mutation were resistant to PLX4032. Seven *BRAF*^V600E ^mutant cell lines were sensitive to PLX4032, including four highly sensitive cell lines with half maximal inhibitory concentration (IC_50_) values below 1 μM. Surprisingly, in three cell lines with *BRAF*^V600E ^mutation we could not determine an IC_50 _with increasing concentrations of PLX4032 up to 10 μM, suggesting that these cell lines are resistant to this agent in a 5-day exposure *in vitro *(Figure 1C). Similar results have been obtained in 3-day viability assays and when PLX4032 is added daily to the cultures or just at the beginning of the experiment (data not shown).

### PLX4032 has similar inhibitory effects on cell cycle in sensitive and resistant *BRAF*^V600E ^mutant cell lines

**Figure 2:**
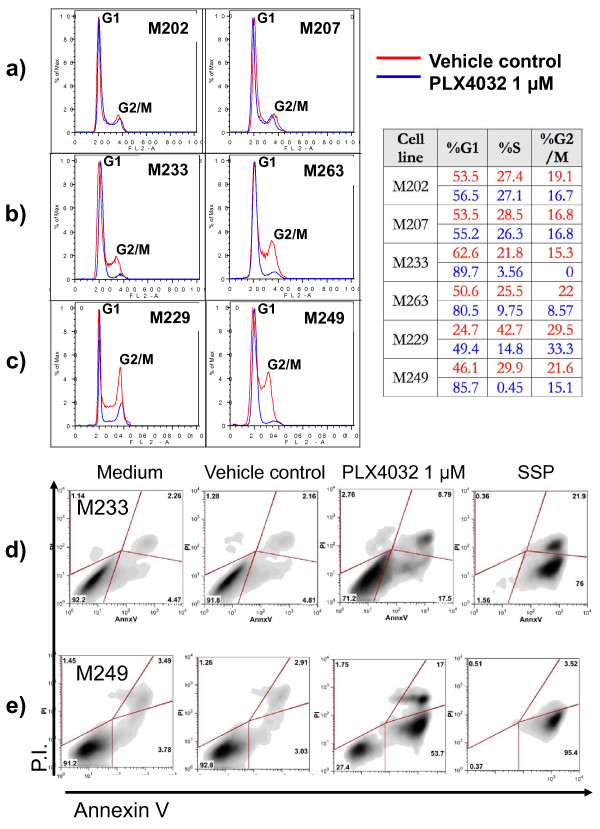
**Effects of PLX4032 on cell cycle and apoptosis**. a-c) Melanoma cell lines were cultured with 1 μM of PLX4032 for 20 hours and stained with propidium iodide for cell cycle analysis gated on live cells. a) *NRAS *Q61L mutants, b) *BRAF*^V600E ^mutants resistant to PLX4032, c) *BRAF*^V600E ^mutants sensitive to PLX4032. d-e) Melanoma cell lines were cultured with 1 μM of PLX4032, vehicle control, or 1 μM of staurosporine (SSP - positive control to induce apoptosis) for 120 hours and analyzed by flow cytometry for apoptotic cell death upon double-staining with Annexin V and propidium iodide. Testing included a PLX4032-resistant cell line (M233) and a highly sensitive cell line (M249), both of which are *BRAF*^V600E ^mutants.

To study effects of PLX4032 on cell cycle progression downstream of B-Raf signaling we used propidium iodide flow cytometric staining. As expected, PLX4032 had no effect on cell cycle progression in melanoma cell lines without a *BRAF*^V600E ^mutation (Figure 2A). In contrast, PLX4032 exposure for one (data not shown) or 20 hours (Figure 2B and 2C) led to a similar and profound G1 arrest in all *BRAF*^V600E ^mutant cell lines regardless of their *in vitro *sensitivity to PLX4032.

### PLX4032 leads to apoptotic death in sensitive *BRAF*^V600E ^but not in resistant *BRAF*^V600E ^mutated melanoma cell lines

We then analyzed the ability of PLX4032 to differentially induce apoptotic effects against melanoma cell lines with the *BRAF*^V600E ^mutation. Using a *BRAF*^V600E ^mutant melanoma cell line with a good response to PLX4032 (M249) and another one that was poorly responsive to PLX4032 (M233) based on cell viability assays, we analyzed apoptotic induction using flow cytometry based on the incorporation of propidium iodide and Annexin V. After PLX4032-treatment, the increase in Annexin V positive cells, with or without being double positive for propidium iodide, was greater in the PLX4032-responsive M249 cells compared to the poorly responding M233 cells (Figure 2D and 2E). Similar results were obtained with M238 and M263 (data not shown). Taken together with the data on cell cycle inhibition, these data suggest that PLX4032 has cytostatic effects in *BRAF*^V600E ^mutant cell lines with a poor response, while it has cytostatic and cytotoxic effects in cell lines with a good response to PLX4032 in cell viability assays.

### Functional and genomic characterization of *BRAF*^V600E ^mutated cell lines with different sensitivity to PLX4032

We tested if the differences in sensitivity to PLX4032 were due to markedly different doubling times. Resistant *BRAF*^V600E ^mutated cell lines tended to have a slower doubling time compared to the sensitive *BRAF*^V600E ^mutated cell lines (P = 0.24, Table 1). The lack of significance was due to outliers in a small group, most notably the highly sensitive cell line M262 having a doubling time close to 50 hours. Interestingly, all cell lines homozygous for the *BRAF*^V600E ^mutation were moderately to highly sensitive to PLX4032, and cell lines resistant to PLX4032 were all heterozygous for *BRAF*^V600E ^(P = 0.16). However, there were also two highly sensitive heterozygous cell lines with IC_50 _values below 1 μM of PLX4032, and the sensitivity of homozygous cell lines spreads through one-log differences in PLX4032 concentrations (Table 1). We then used high throughput analysis of over 500 gene mutations using mass-spectrometry based genotyping [11] and high-density SNP arrays to explore other genomic alterations. Two different platforms (Illumina and Affymetrix) gave highly concordant results (data not shown) demonstrating that out of the 10 cell lines with *BRAF*^V600E ^mutation, four have amplification of the *BRAF *locus (Table 1). There was no clear relationship between these amplification events and the *BRAF*^V600E ^zygosity or the sensitivity to PLX4032. There were very few secondary mutations in this group of cell lines, with one cell line having a mutation in *EGFR*, and one cell line with a mutation in *AKT *(Table 1). In addition, the M257 cell line, which is wild type for both *NRAS *and *BRAF *and is highly resistant to PLX4032, was found to have 3 copies of wild type *BRAF *and a point mutation in *CDKN2A*. The distribution of amplification events in *MITF *and *EGFR *were also spread among the cell lines. Of note, there was no clear trend regarding the activation of the PI3K/Akt pathway based on activating mutations, or amplifications of *AKT1/2 *segregating the resistant and sensitive cell lines. Supervised hierarchical clustering comparing SNP array data from PLX4032-resistant and -sensitive *BRAF*^V600E ^mutant cell lines did not point to specific genomic areas with concordant alterations differentiating the two groups of cell lines.

### Modulation of MAPK and PI3k/Akt signaling pathways in sensitive and resistant cell lines

**Figure 3:**
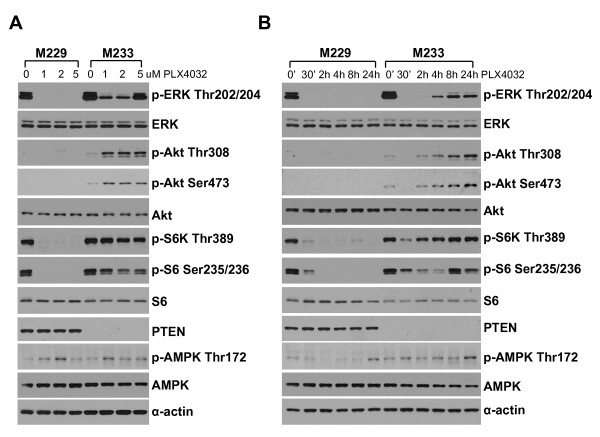
**Western blot analysis of phosphorylated and total amount of key proteins in the MAPK and PI3k/Akt pathways**. a) The PLX4032-sensitive M229 cell line and the PLX4032-resistant M233 cell line were cultured in different concentrations of PLX4032 for 24 hours and lysates were analyzed by Western blot. b) M229 and M233 cells were treated by PLX4032 in a time course over 24 hours, and cell lysates were analyzed by Western blot.

To further explore how cell lines with *BRAF*^V600E ^mutation respond differently to PLX4032 we chose two extreme examples of cell lines with similar growth kinetics to perform an extended analysis of signaling pathways (Figure 3). M229 is one of the two most sensitive cell lines, while M233 proved to be very resistant despite having a short *in vitro *doubling time (Table 1). Exposure to PLX4032 resulted in a marked decrease in pErk in both cell lines, but this was more prominent and durable in the sensitive M229 compared to the resistant M233. M229 has a heterozygous *PTEN *deletion by SNP array analysis, and had a detectable band for PTEN protein by Western blot that did not change with PLX4032 exposure. The resistant M233 cell line has a homozygous *PTEN *deletion and has no PTEN protein by Western blot. This correlates with baseline pAkt detectable in M233 but not M229, as well as increase in pAkt upon PLX4032-exposure in the resistant M233 but not in the sensitive M229 cell line. Interestingly, pS6 decreased in both cell lines upon PLX4032 exposure. Finally, we explored if there was modulation of AMPK, which has been recently described as a downstream modulator of glucose metabolism in *BRAF*^V600E ^mutants [20]. There was a low level of induction of pAMPK. These studies demonstrate that PLX4032 has complex effects on MAPK and PI3k/Akt signaling pathways that may be dependent on secondary oncogenic events beyond B-Raf.

### Non-invasive imaging of PLX4032 anti-tumor activity

**Figure 4:**
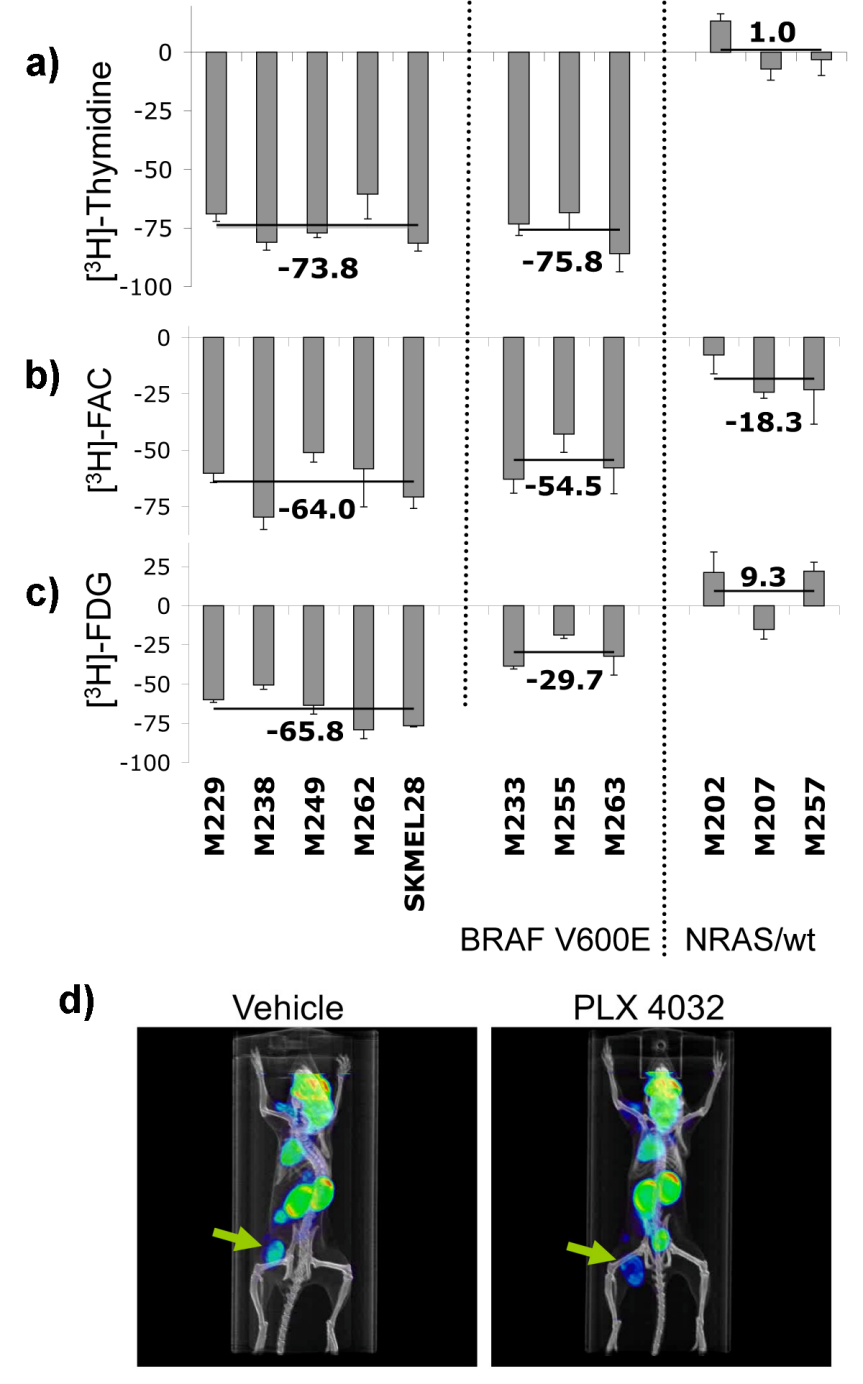
**Metabolic tracer uptake profile upon exposure to PLX4032**. a-c) *in vitro *PET tracer uptake profiles for 11 different melanoma cell lines. Tritium counts was measured on a micro-beta reader and PLX4032 treated cells were compared to vehicle control and relative PET tracer uptake calculated. a) [^3^H]-thymidine uptake profile, b) [^3^H]-FAC uptake profile, c) [^3^H]-FDG uptake profile. The black lines and the number next to them represent the average change in PET tracer uptake of the cell lines with the same mutational status and sensitivity towards PLX4032. d) [^18^F]FDG PET tracer uptake *in vivo*. SCID/beige mice with 5-7 mm M249 melanoma xenografts on the left lower flank were treated twice daily with 100 mg/kg of PLX4032 or vehicle control by oral gavage. Three days later mice were imaged by microPET scanning upon administration of [^18^F]-FDG.

We analyzed the uptake profile of three different metabolic tracers that can be used in PET scans: two nucleoside analogs (thymidine and FAC [21]) and FDG, a glucose analog widely used as a PET tracer. As expected, *BRAF *wild type cell lines had no significant change in uptake of thymidine or FAC upon PLX4032-exposure. Conversely, all *BRAF*^V600E ^mutated cell lines, irrespective of their sensitivity to PLX4032, had markedly decreased uptake of these two nucleoside analogues (Figure 4a and 4b). The greatest difference between PLX4032-sensitive and -resistant *BRAF*^V600E ^mutants was in FDG uptake. The percentage decrease in FDG uptake was roughly double in PLX4032-sensitive *BRAF*^V600E ^mutants compared to PLX4032-resistant cell lines (P = 0.009, Figure 4c). Finally, we tested if [^18^F]-FDG uptake could be used as a pharmacodynamic marker of B-Raf^V600E ^inhibition by PLX4032 *in vivo*. Mice with established subcutaneous M249 melanoma xenografts, a cell line highly sensitive to PLX4032 *in vitro*, were treated for 3 days with PLX4032 twice daily by oral gavage, and then analyzed by combined microPET and microCT using [^18^F]-FDG as PET tracer. There was a 32% decrease in [^18^F]-FDG uptake compared to the vehicle control mice, even though tumor sizes were not different at this early time point (Figure 4d). In conclusion, inhibition of [^18^F]-FDG uptake can be used as an early marker of effective B-Raf^V600E ^inhibition by PLX4032.

## Discussion

The *BRAF*^V600E ^mutation is one of the most common kinase domain mutations in human cancer with a particularly high incidence in malignant melanoma [3,7]. The Raf-inhibitors PLX4720 and PLX4032 have the preclinical characteristics of functioning as specific inhibitors of the *BRAF*^V600E ^mutated kinase with a favorable profile compared to wild type kinases [9,22]. Understanding the patterns of sensitivity and resistance in melanomas with different oncogenic events is of high importance for clinical translation. Our studies confirmed the high specificity of PLX4032 for a subset of *BRAF*^V600E ^mutant cell lines [22]. Surprisingly, we noted differences in the sensitivity to PLX4032, with some *BRAF*^V600E ^mutants demonstrating resistance to the cytotoxic effects of PLX4032. In most cases, these cells had a tendency towards slower growth kinetics and being heterozygous for *BRAF*^V600E^.

This differential response to PLX4032 in *BRAF*^V600E ^mutant melanoma cell lines may be explained by several mechanisms. It may be that there is preferential MAPK pathway-addiction in sensitive cell lines, and cells with lower sensitivity are less dependent on the *BRAF*^V600E ^oncogenic signaling, relying on the co-activation of other signaling pathways including the PI3K/Akt pathway. We explored this possibility with SNP arrays and high throughput oncogene sequencing with a particular interest in looking at this pathway. The genomic analysis revealed that alterations in PI3K/Akt, including deletions of *PTEN*, amplifications of *AKT *and activating mutations in *AKT *were distributed throughout the cell line list with no clear pattern of correlation with sensitivity or resistance to PLX4032. However, in two cell lines phospho-specific Western blot staining suggested that the resistant cell line had increased Akt signaling upon PLX4032 exposure. Another possibility is that PLX4032-resistant *BRAF*^V600E ^mutants have alternative signaling at the level of Raf, as has been described for cell lines with acquired resistance to a different Raf-inhibitor, AZ628, which show increased signaling through C-Raf [23]. The increase in pErk in an *NRAS *Q61L mutant cell line could be explained by abrogation of negative feedback loops mediated mainly by dual specificity phosphatases (MKPs/DUSPs), as reported with Mek inhibitors [17,24], and the recent description of increased C-Raf signaling when heterodimerizing with inhibited B-Raf in *BRAF *wild type cells [18,19]. Therefore, the modulation of feed-back loops and alteration of Raf dimerization upon treatment with Raf inhibitors may also have a role in the differential sensitivity to PLX4032 in *BRAF*^V600E ^mutant cell lines. Finally, differences in expression of pro- and anti-apoptotic molecules like Bim and Bad [25] may allow some *BRAF*^V600E ^mutant cell lines to undergo growth arrest but not die from apoptosis upon exposure to PLX4032. Studies are ongoing to further explore these possibilities.

We explored the use of PET imaging as a mean to non-invasively detect PLX4032-sensitivity. *In vitro *we found that any of the three PET tracers FDG, FLT and FAC could be used to distinguish between melanomas with a *NRAS *or a *BRAF*^V600E ^mutation based on the differential effects of PLX4032 on cell cycle and metabolism. FDG could furthermore be used to distinguish between *BRAF*^V600E ^mutant melanomas with high or low sensitivity to PLX4032. The PI3K/Akt pathway has been widely regarded as having a role in the regulation of glucose metabolism through mTOR, but recently the LKB1-AMPK pathway has been found to be regulated by oncogenic *BRAF*^V600E ^signaling [20], which together may explain the marked and rapid effects of PLX4032 on inhibiting FDG uptake. We explored this possibility in two cell lines. Our data suggests a minor increase in pAMPK upon PLX4032 exposure, which may be in line with the proposed hypothesis [20].

## Conclusions

These studies in melanoma cell lines may allow to better interpret the results of a recently reported phase I clinical trial with PLX4032 [26], with an objective response in excess of 70% of patients with *BRAF*^V600E ^positive metastatic melanoma. The characterization of PLX4032-sensitive and -resistant *BRAF*^V600E ^mutant melanoma cell lines may provide information about the molecular mechanisms that dictate sensitivity and resistance to PLX4032. In addition, molecular imaging with [^18^F]FDG PET scans may help in providing an early readout of complete or incomplete pharmacodynamic effects of PLX4032 and therefore predict lesions that may or may not respond to therapy.

## Abbreviations

(BRK): Breast tumor kinase; (MKPs/DUSPs): Dual specificity phosphatases; (FDG): 2-fluoro-2-deoxy-D-glucose; (FAC): 2'-Deoxy-2'-fluoroarabinofuranosylcytosine-[^3^H]; (MTA): Materials transfer agreement; (MTS): 3-(4,5-dimethylthiazol-2-yl)-5-(3-carboxymethoxyphenyl)-2-(4-sulfophenyl)-2H-tetrazolium; (IC_50_): Half maximal inhibitory concentration; (MAPK): Mitogen-activated protein kinase; (pErk): Phosphorylated Erk; (PET): Positron emission tomography; (thymidine): Thymidine [methyl-^3^H]; (UCLA): University of California Los Angeles.

## Competing interests

The authors declare that they have no competing interests.

## Authors' contributions

JNS, RN, QW, DG, TH, SM, HS, LEM, JGB, SK, NA, EVE, JZ, BC, BAC, RCK: Performed experiments.

JNS, PMRSL, AR: Planned the studies and wrote the manuscript.

All authors have read and approved the final manuscript.
